# Improving consensus contact prediction via server correlation reduction

**DOI:** 10.1186/1472-6807-9-28

**Published:** 2009-05-06

**Authors:** Xin Gao, Dongbo Bu, Jinbo Xu, Ming Li

**Affiliations:** 1 David R. Cheriton School of Computer Science, University of Waterloo, N2L3G1, Canada; 2 Institute of Computing Technology, Chinese Academy of Sciences, Beijing, 100080, PR China; 3 Toyota Technological Institute at Chicago, 1427 East 60th Street, Chicago, Illinois 60637, USA

## Abstract

**Background:**

Protein inter-residue contacts play a crucial role in the determination and prediction of protein structures. Previous studies on contact prediction indicate that although template-based consensus methods outperform sequence-based methods on targets with typical templates, such consensus methods perform poorly on new fold targets. However, we find out that even for new fold targets, the models generated by threading programs can contain many true contacts. The challenge is how to identify them.

**Results:**

In this paper, we develop an integer linear programming model for consensus contact prediction. In contrast to the simple majority voting method assuming that all the individual servers are equally important and independent, the newly developed method evaluates their correlation by using maximum likelihood estimation and extracts independent latent servers from them by using principal component analysis. An integer linear programming method is then applied to assign a weight to each latent server to maximize the difference between true contacts and false ones. The proposed method is tested on the CASP7 data set. If the top *L*/5 predicted contacts are evaluated where *L *is the protein size, the average accuracy is 73%, which is much higher than that of any previously reported study. Moreover, if only the 15 new fold CASP7 targets are considered, our method achieves an average accuracy of 37%, which is much better than that of the majority voting method, SVM-LOMETS, SVM-SEQ, and SAM-T06. These methods demonstrate an average accuracy of 13.0%, 10.8%, 25.8% and 21.2%, respectively.

**Conclusion:**

Reducing server correlation and optimally combining independent latent servers show a significant improvement over the traditional consensus methods. This approach can hopefully provide a powerful tool for protein structure refinement and prediction use.

## Background

Computational protein structure prediction has made great progress in the last three decades [1,2]. Protein inter-residue contact prediction is one of the problems being actively studied in the structure prediction community. Recent CASP (Critical Assessment of Techniques for Protein Structure Prediction) [3-7] events have demonstrated that a few true contacts, extracted from template-based models, can provide very important information for protein structure refinement, especially on targets without good templates in PDB [8]. For example, Misura *et al*. [9] have revised the widely-used *ab initio *folding program, Rosetta [10], by incorporating inter-residue contact information as a component of Rosetta's energy function, and shown that the revised Rosetta exhibits not only a better computational efficiency, but also a better prediction accuracy. For some test proteins, the models built by this revised Rosetta are more accurate than their template-based counterparts, which is rarely seen before [7]. Zhang-server [11] and TASSER [12] perform very well in both CASP7 and CASP8. One of the major advantages of these two programs over the others is that both depend on contacts and distance restraints, extracted from multiple templates, to refine the template-based models. It has been shown by Zhang *et al*. that *ab initio *prediction methods can benefit from contact predictions with an accuracy that is higher than 22% [13].

Protein inter-residue contact was first studied by [14-17] to calculate the mean force potential. Göbel *et al*. [18] formally proposed the problem of contact prediction, and showed that correlated mutation (CM) is useful information to predict inter-residue contacts. The fundamental assumption is that if two residues are in contact with each other, during evolution, if one residue mutates, the other one has a high chance to mutate as well. Thus, by analyzing residue mutation information from multiple sequence alignments, it can be predicted whether or not two residues are in contact. Since then, different correlated mutation statistical methods have been carefully examined [19-24].

According to whether structural templates information is taken into consideration, contact prediction methods can be classified into two categories: sequence-based methods and template-based methods. Among all the sequence-based methods, some rely solely on correlated mutation information calculated by different statistical approaches [18,22-24], while others encode the correlated mutation, together with other features such as secondary structure and solvent accessibility, into machine learning models [25-32]. Although the correlated mutation performs well on local contact prediction, which is usually defined to be two residues within six amino acids from each other in the protein sequence, it usually fails for non-local contacts. Therefore, other information such as evolutionary information and secondary structure information, has been applied to improve the performance of contact prediction methods [25-33]. In [25], Fariselli *et al*. encoded four types of features into a neural network based server (CORNET): 1) correlated mutation, 2) evolutionary information, 3) sequence conservation, and 4) predicted secondary structure.

They defined that two residues are in contact if the Euclidean distance between the coordinates of their *C*_*β *_atoms (*C*_*α *_atom for Glycine) is smaller than 8Å, and the sequence separation between the two residues is at least seven to eliminate the influence of local *α*-helical contacts. CORNET achieves an average accuracy of 21%. Other features have been investigated since then [27,29]. PROFcon [31], one of the best three contact prediction servers in CASP6 [5], encodes more information into its neural network model, including solvent accessibility and secondary structure over the regions between the two residues, as well as the properties of the entire protein. PROFcon performs impressively on small proteins or alpha/beta proteins with an accuracy of more than 30%. Recently, Shackelford and Karplus [33] proposed a neural network based method to calculate the correlated mutation by using the statistical significance of the mutual information between the columns of multiple sequence alignment. Their SAM-T06 server outperforms all the other contact prediction servers in CASP7, and achieves an average accuracy of 45% for all CASP7 target proteins, which is higher than that of any previously reported study.

In contrast to these sequence-based methods, which encode correlated mutation information and other sequence-derived information, there are some studies on predicting inter-residue contacts from structural templates [9,32,34-36]. The underlying assumption for such methods is that contacts are usually very conserved during evolution. Consequently, templates, with structures similar to that of the target protein, usually contain common contacts, such that consensus methods work well. Bystroff *et al*. [34,35] have considered folding pathways, and predicted contacts by employing HMMSTR [37], a hidden Markov model for local sequence-structure correlation. LOMETS [36], a majority voting based consensus method, takes nine state-of-the-art threading programs as inputs. LOMETS predicts contacts by attempting to select the best input model.

Recently, two Support Vector Machines (SVMs) based contact prediction methods, SVM-SEQ and SVM-LOMETS, have been proposed by Wu *et al*. [32]. SVM-SEQ only takes sequence-derived information into consideration, whereas SVM-LOMETS, a consensus method, is based on structural templates. SVM-LOMETS differs from its ancestor, LOMETS, in that it carefully trains contact frequency, *C*_*α *_distances, and template quality by an SVM model. The inputs for SVM-LOMETS are nine state-of-the-art threading programs: FUGUE [38], HHSEARCH [39], PAINT, PPA-I, PPA-II [36], PROSPECT2 [40], SAM-T02 [41], SP3 and SPARKS2 [42]. Both SVM-SEQ and SVM-LOMETS are tested on a set of 554 proteins, on which each achieves an average accuracy of 29% and 53%, respectively. Although it is widely acknowledged that a method usually has different performance on different data sets, one can still expect that a consensus contact prediction method will outperform the individual servers. Instead of testing on the entire CASP7 data set, these two programs are further tested on the 15 new fold (NF) targets of CASP7. The average accuracies are 26% and 11%, respectively. Through a comprehensive comparison of sequence-based and structure-based methods, including SVM-SEQ, SVMCON [43], SVM-LOMETS, LOMETS, and SAM-T06 server, Wu *et al*. have concluded that template-based methods are better than sequence-based methods on template-based modeling (TBM) targets, but worse on new fold targets. However, even for new fold targets, where the threading methods fail to identify good templates, the templates discovered by threading usually contain many true contacts. The major challenge is that some true contacts are not contained in a majority of the top templates, resulting in the failure of the traditional majority voting methods. In this paper, we propose a novel consensus contact prediction method to eliminate the effect of server correlation, and to discover true contacts even when they are not commonly found in the top templates. All the contacts, determined by structure prediction servers, are considered to be candidates. Our consensus method then assigns a confidence score to each contact candidate, while also taking correlated mutation information into consideration.

## Results

### Data Set

#### Server Selection

To evaluate the performance of the proposed consensus method, six threading-based protein structure prediction servers are used: FOLDpro [44], mGenThreader [45,46], RAPTOR [47,48], FUGUE3 [38], SAM-T02 [41], and SPARK3 [42]. Although there are some servers, such as Rosetta and Zhang-server, with a better performance than that of the six servers, they are not used because their models are already refined by predicted contacts.

#### Training and Test Data

The biennial CASP competition provides us a comprehensive and objective data set. The CASP7 targets and models generated by the six servers are adopted as the training and test data. For each server on a target, the five submitted models are considered. All server models are downloaded from the CASP7 website, except for mGenThreader, which does not participate in CASP7. We submitted the CASP7 targets to the mGenThreader web server, and downloaded models from there before August 2006. Therefore, all these models are generated before the native structures of the CASP7 targets are released. Eighty nine CASP7 target proteins are used as valid targets for the CASP7 evaluation, while 104 protein sequences are released as targets. Redundancy is removed at the 40% sequence identity level by using CD-HIT [49], which results in 88 target proteins. Only T0346 is removed, because it shares 71% sequence identity with T0290. Furthermore, two targets (T0334 and T0385) are removed from the data set due to some errors in the models, generated by some of the six individual servers on these two targets; for example, the models generated by some servers only cover discontinued regions of the target proteins. To conduct a cross-validation, the 86 target proteins are randomly divided into four sets of 22, 21, 21, and 22 proteins, respectively. If one target belongs to a certain set, all of its models and contacts are in the same set.

#### Data Set Statistics

The performance of the six individual servers are compared in terms of prediction accuracy and coverage. In evaluating the performance of a server, only the best models of each target are considered. If the number of contacts in a model is less than *L*/5, where *L *is the target size, both the accuracy and the coverage for this model are set to 0. As shown in Table 1, the average accuracy of the six servers ranges from 43% to 53%. The SAM-T02 server has the highest accuracy but the lowest coverage. The artificial server "Overall" in Table 1 means a server that generates the union set of all contacts contained in the best models. The accuracy of server "Overall" is very low (12%), compared to that of any individual server. Note that the server "Overall" consistently contains many more true contacts than any individual server does. Therefore, the low accuracy of the server "Overall" implies that the false contacts, generated by these individual servers, differ from each other in most cases, whereas the individual servers tend to generate common true contacts. This means the consensus method can probably be employed to differentiate true contacts from false ones.

**Table 1:** Average and deviation of contact accuracy and coverage of the six individual servers on the 86 CASP7 targets

	*Accuracy*		*Coverage*	
	
	*Average*	*Deviation*	*Average*	*Deviation*
FOLDpro	45	8.2	48	9.3
mGenThreader	43	6.6	45	8.5
RAPTOR	48	6.6	52	7.0
FUGUE3	46	7.9	37	5.5
SAM-T02	53	6.5	37	5.5
SPARK3	48	7.3	51	7.6

Overall	12	7.2	80	2.3

All values are percentiles.

As shown in Table 1, the average coverage of the six servers ranges from 37% to 52%. However, when they are combined, the coverage for server "Overall" is very high (approximately 80%). This indicates that some true contacts are predicted by only a small number of individual servers, and the different servers can predict a common subset of the true contacts. On the other hand, this implies that most of the native contacts are contained in the models, generated by threading programs. Thus, the challenge is how to identify them.

### Server Correlation and Latent Servers

This section studies the correlation of the six individual servers and derives the latent servers. Table 2 lists the pairwise correlation of the six individual servers, which is calculated according to Eq. (3). Note that the matrix is not symmetric, because different servers predict different numbers of contacts. As shown in Table 2, the correlation between two different servers ranges from 0.25 to 0.59, which implies that some servers are more closely correlated than others in terms of contact prediction. Therefore, the majority voting based consensus methods, which simply apply majority voting and assume each server is independent, will not always work because some of the common components of these servers are over-expressed.

**Table 2:** Pairwise correlation of the six individual servers

Server	FOLDpro	mGenThreader	RAPTOR	FUGUE3	SAM-T02	SPARK3
FOLDpro	1	0.34	0.43	0.25	0.30	0.41
mGenThreader	0.35	1	0.42	0.26	0.30	0.41
RAPTOR	0.43	0.41	1	0.30	0.35	0.51
FUGUE3	0.35	0.35	0.40	1	0.37	0.40
SAM-T02	0.50	0.50	0.59	0.47	1	0.59
SPARK3	0.40	0.41	0.50	0.29	0.34	1

The relationship between the latent servers and the individual ones is then derived according to Eq. (4). Note here that the top five models for each target of each server are considered. The confidence score of a server on a contact candidate is estimated by the number of models in the top five, containing this contact, divided by the total number of models considered (five in this case). As shown in Table 3, different latent servers represent different individual servers. For example, *H*_1 _represents the common characteristics shared by these individual servers, because the weights of *H*_1_, on these individual servers, are about the same; *H*_2 _differentiates FUGUE3 from the other servers; *H*_3 _represents FOLDpro by a large positive weight, and represents mGenThreader by a large negative weight. Based on the eigenvalues, *H*_6 _is eliminated, since the eigenvalue for *H*_6 _is much smaller than that of the others. Thus, *H*_6 _is considered as random noise.

**Table 3:** Relationship among the six individual servers and the independent latent servers

Server	H1	H2	H3	H4	H5	H6
FOLDpro	0.37	-0.35	0.66	0.01	-0.08	-0.55
mGenThreader	0.37	-0.26	-0.75	-0.01	-0.02	-0.48
RAPTOR	0.42	-0.23	0.04	0.27	0.76	0.36
FUGUE3	0.37	0.82	0.04	0.37	0.01	-0.22
SAM-T02	0.49	0.20	0.03	-0.81	-0.04	0.23
SPARK3	0.41	-0.21	-0.02	0.36	-0.65	0.49

The optimal weights for the latent servers are derived by the cross-validation of the four sets. Correlated mutation is considered to be another independent latent server, because it provides a target sequence-related probability for each contact candidate. Correlated mutation is calculated as previously described in [18,22]. Each time the ILP model is trained on three of the four sets, and a set of weights is optimized by the ILP model, based on which a new prediction server is derived, named as , and , respectively. In this paper, S* refers to server  on test set *i *(*i *= 1, 2, 3, 4). Since the inputs are the six individual servers, after the optimal weights λ* are calculated by the ILP model, each hidden server in Eq. (1) are further replaced by the linear combination of the original individual servers as calculated in Eq. (5). Table 4 shows the linear combination representation of S* on the individual servers and correlated mutation. It is clear that the four sets of weights are very similar. Note that mGenThreader has negative weights. This implies that the contribution of mGenThreader is accounted for by the other individual servers.

**Table 4:** Linear combination representation of new server S* on the six individual servers and correlated mutation

S	FOLDpro	mGenThreader	RAPTOR	FUGUE3	SAM-T02	SPARK3	CM
	0.29	-0.28	1.27	1.47	0.23	0.62	0.30
	0.301	-0.27	1.35	1.35	0.22	0.58	0.37
	0.38	-0.29	1.37	1.36	0.14	0.65	0.28
	0.29	-0.44	1.29	1.39	0.12	0.56	0.23

CM: correlated mutation.

### CASP7 Evaluation

**Figure 1:**
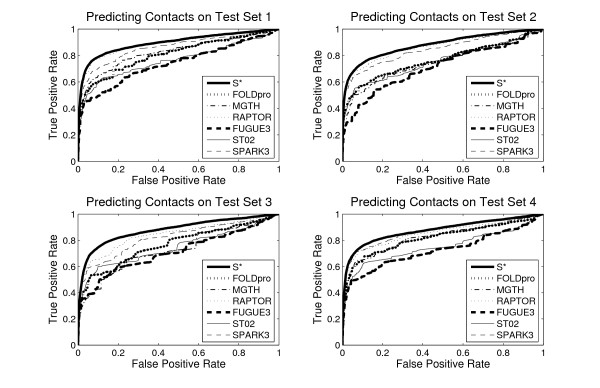
**ROC curves for our method and the six individual servers**. Performance comparison using ROC plots for S* (thick solid line), FOLDpro (thick dotted line), mGenThreader (thin dashdot line), RAPTOR (thin dotted line), FUGUE3 (thick dashed line), SAM-T02 (thin solid line), and SPARK3 (thin dashed line).

We first assess our consensus server S* by Receiver Operating Characteristic (ROC) plots. They provide an intuitive way to examine the trade-off between the ability of a classifier to correctly identify positive cases and to incorrectly classify negative cases. Figure 1 depicts the performance of server S* and the six individual servers on the four test sets.

As shown in Figure 1, server S* performs better than any individual server on all the four test sets. For each server, the performance of this server on test set 1 is slightly better than that on the other three test sets, which means test set 1 is the easiest among those four. RAPTOR performs better than other individual servers on the first three test sets, and SPARK3 exhibits the best performance on test set 4. There are distinct performance differences between server S* and the best individual server on test set 1, 2, and 4, when the false positive rate is below 0.3. However, the difference is not obvious on those three test sets, when the false positive rate is higher than 0.3. For test set 3, the most difficult test set, the performance of S* is much better than that of any individual server all the time. It is also noticeable that the curve of S* is much smoother than that of the individual servers.

Then, the accuracy of S* is evaluated. Table 5 summarizes the average accuracy of S* and the majority voting method on the four test sets, where different numbers of top contacts are evaluated. Recall S* generates a confidence score for each contact candidate. The top contacts for each target are readily found by sorting the candidates according to their confidence scores. The majority voting method is implemented as follows: for each contact candidate, its confidence score by the majority voting method is calculated as the sum of the confidence scores assigned by the six servers. After the scores of all the contact candidates are calculated and sorted, different numbers of the top candidates are chosen.

**Table 5:** Average accuracy of the top contacts predicted by S* on different test sets, and the accuracy of the majority voting method

# Contacts	*Accu*_*set*1_	*Accu*_*set*2_	*Accu*_*set*3_	*Accu*_*set*4_	*Accu*_*overall*_	*Accu*_*mv*_
*L*	69	60	57	65	**63**	**61**
*L*/2	75	67	63	72	**69**	**66**
*L*/5	80	73	67	74	**73**	**68**
*L*/10	79	74	69	76	**75**	**71**

The first column shows the number of top contacts being considered. The second to fifth columns show the accuracy of our method on the four test sets respectively. The sixth column shows the overall accuracy of our method on all the four test sets. The last column shows the overall accuracy of the majority voting method on all the four test sets. All values are percentiles.

**Figure 2:**
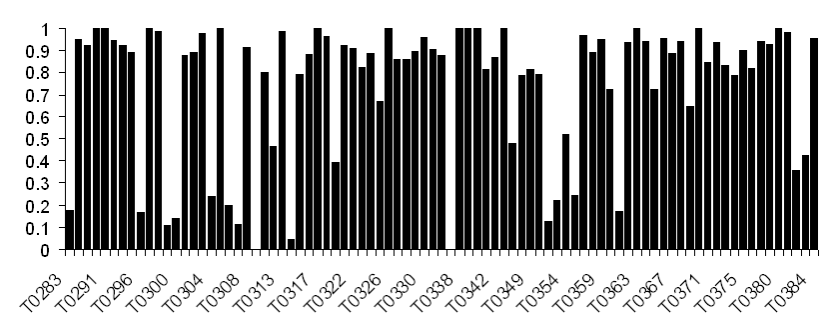
Prediction accuracy for the top *L*/5 contacts of S* on each CASP7 target.

As shown in Table 5, the average accuracy increases when the number of the top contacts decreases, except for server S* on test set 1, in which the accuracy for the top *L*/10 contacts is slightly lower than that for the top *L*/5 contacts. This occurs because *L*/10 is usually a small number (20–30 for most cases), and a few incorrectly predicted top contacts will influence the total accuracy significantly. The overall accuracy of S* on all the four test sets is at least 63%, and is consistently higher than the accuracy of the majority voting method. For the top *L*/5 contacts, the accuracy of S* is 73%, which is about 5% higher than that of the majority voting method, and much higher than the accuracy of any previously reported study. Figure 2 reflects the prediction accuracy for the top *L*/5 contacts of S* on each CASP7 target. It can be seen that the accuracy is higher than 80% on most targets. In fact, of the total 86 targets, S* has an accuracy of 100% on 13 targets, higher than 90% on 39 targets, higher than 80% on 58 targets, and below 40% on only 16 targets. Note that S* has an accuracy of 0 on two targets: T0309 (free modeling target) and T0335 (template based modeling target). We carefully look into these two cases. Both targets are very short. The target sequences, published by CASP7 for T0309 and T0335, have 76 and 85 residues, respectively. However, the experimentally determined size used by CASP7 to evaluate these two targets are only 62 and 36, respectively. This conveys that some parts of the targets are not experimentally determinable nor accurate enough. Thus, *L*/5 is only 12 and 7 for the two targets. Additionally, all the six individual servers perform poorly in terms of the contact prediction, which means there are only a few correct candidates among a large number of incorrect ones. This can explain the failure of S* on T0309 and T0335.

To evaluate more carefully how much our consensus method can improve upon individual servers and the simple majority voting method, all the targets are divided into three categories: easy (high accuracy), medium (template based modeling), and hard (new fold), according to the CASP7 assessment . Table 6 shows the average accuracy and deviation on the top *L*/5 contacts of S*, the six individual servers, and the majority voting method. As shown in Table 6, for easy, medium, and hard targets, the accuracy of S* on the top *L*/5 contacts is 94%, 76%, and 37%, respectively, and much higher than the best individual server, where the improvement is at least 17% for each case. On the other hand, server S* always performs better than the majority voting method, and the improvements are about 2%, 5%, and 24%, respectively. This exactly verifies the server correlation assumption because, for easy targets, individual servers usually do well, which means for a contact candidate, the more servers that support it, the more likely it is correct. However, the majority voting rule does not always work on medium and hard targets, because it suffers from the over-expressed common components of the input servers due to the server correlation. Thus, our consensus method does much better than the majority voting method on harder targets.

**Table 6:** Accuracy and deviation of top *L*/5 contacts of the six individual servers, the majority voting method, and our method on easy, medium, and hard target sets

Server Name	Easy Targets		Medium Targets		Hard Targets	
	
	*Accu*.	*Dev*.	*Accu*.	*Dev*.	*Accu*.	*Dev*.
FOLDpro	**77**	1.1	44	5.3	10	5.8
mGenThreader	68	3.8	43	4.4	11	7.4
RAPTOR	75	1.3	50	3.9	13	7.1
FUGUE3	75	0.7	47	6.2	12	9.1
SAM-T02	75	1.3	**54**	5.2	**17**	14.7
SPARK3	76	1.4	48	4.7	11	7.4
Majority Voting	**92**	0.7	**71**	8.1	**13**	6.9
S*	**94**	0.4	**76**	8.5	**37**	28.2

Accu.: Accuracy. Dev.: Deviation. All values are percentiles.

Depending on the sequence separation, contacts can be classified as short-range contacts (separation 6–11), medium-range contacts (separation 12–24), and long-range contacts (separation >24) [32,43]. The performance of our method is further evaluated on different separation ranges for target protein classes with different difficulty levels. As shown in Table 7, the accuracy of a certain separation range decreases clearly when target proteins become harder. For easy targets, the accuracy of long-range contacts is higher than that of short- and medium-range contacts. This makes sense because for an easy target, it is very likely that all the individual servers predict models that have very similar topology to the native structure. Thus, these models contain common long-range contacts, which helps to determine the overall topology. For medium targets, our method achieves similar performance on different separation ranges. Not surprisingly, when applied on hard targets, the accuracy of long-range contacts is much worse than that of short- and medium-range contacts. This coincides with the fact that the individual servers are usually not able to generate models with correct folds, which causes most long-range contact candidates to be wrong ones. Among the three categories of the test proteins, the new fold category is much more important than the other two for fairly evaluating the performance of a contact predictor, especially for template-based consensus methods. In fact, new fold targets are adopted as the assessment data set for the contact predictors by CASPs. Table 8 shows the average accuracy on the top *L*/5 contacts of the six individual servers, the majority voting method, and our method on the 15 new fold targets of CASP7. Note that the classification of the new fold targets comes from the assessors of CASP7, according to the criterion that no server could find the correct templates although there might be homologs in the PDB. Our method significantly outperforms the best individual server on eight out of the 15 targets, and performs worse than the best individual server on five targets. It is noticeable that SAM-T02 outperforms our method on four of these five targets. The reason is that SAM-T02 does not generate complete models for these targets. Instead, it generates structures only for some very conserved regions of the targets. The contacts predicted by SAM-T02 thus cover only a small portion of the targets. It can also be seen from Table 8 that our method performs much better than the majority voting method on new fold targets. More specifically, the accuracy of our method at least doubles that of the majority voting method on 10 of the 15 targets. On the other hand, only four of these 15 new fold targets lacked any homologs during CASP7 season, *i.e*., T0287, T0309, T0314, and T0353 [43]. This implies that although other new fold targets have similar structures in the PDB, almost all structure prediction servers fail to detect them. Thus, by using our predicted contacts, one may be able to identify the similar structures for these target proteins, especially for the proteins on which our method achieves a high accuracy, such as T0316_D2, T0319, T0347_D2, T0350, T0356_D1, T0356_D3, and T0386.

**Table 7:** Performance of our method on different separation ranges of target protein classes with different difficulty levels

Target Classes	Short-range	Medium-range	Long-range	All-range
Easy Targets	91	90	93	94
Medium Targets	73	74	70	76
Hard Targets	41	35	26	37
All Targets	72	71	68	73

Top *L*/5 contacts are considered. All values are percentiles.

**Table 8:** Accuracy of top *L*/5 contacts of the six individual servers, the majority voting method, and our method on the 15 new fold targets of CASP7

	FDP	MGTH	RAP	FUG	SAM	SP3	MV	S*
*T*0287	8	6	9	5	12	6	9	33
*T*0296	2	25	7	3	29	7	8	17
*T*0300	16	4	6	10	3	6	8	18
*T*0307	3	6	10	15	18	10	7	12
*T*0309	22	3	6	6	32	5	8	0
*T*0314	12	6	7	8	3	6	8	5
*T*0316_*D*2	15	18	14	24	31	16	21	88
*T*0319	9	9	15	29	0	8	11	40
*T*0347_*D*2	13	3	14	5	46	28	26	48
*T*0350	11	8	27	9	35	26	21	80
*T*0353	17	23	24	29	26	18	12	22
*T*0356_*D*1	4	18	6	3	0	5	8	36
*T*0356_*D*3	6	10	12	9	12	10	11	79
*T*0361	4	4	19	4	9	10	6	21
*T*0386	12	15	23	18	5	11	25	56

*Average*	10	11	13	12	17	11	13	37

FDP: FOLDpro, MGTH: mGenThreader, RAP: RAPTOR, FUG: FUGUE3, SAM: SAM-T02, SP3:

SPARK3, MV: majority voting, S*: our method. All values are percentiles.

We are not able to obtain top-notch contact predictors, such as SVM-LOMETS, the best published consensus method, SVM-SEQ, the best reported study on new fold targets, and SAM-T06 server, the best evaluated contact predictor on CASP7. Thus, the performance of these three methods is retrieved from [32]. When SVM-LOMETS, SVM-SEQ, and SAM-T06 server are applied to the 15 new fold targets of CASP7, each achieves an accuracy of 10.8%, 25.8%, and 21.2% on the top *L*/5 contact predictions, respectively. On the same data set, the accuracy of our method for the top *L*/5 contacts is 37%, which indicates that the improvements are significant. Recall that among all three methods, SVM-LOMETS is the only template-based consensus method. Although the input threading programs of our method are not the same as SVM-LOMETS, both methods contain some common input servers such as FUGUE and SAM-T02. The different input servers are within a similar range of accuracy in terms of structure prediction according to the CASP7 evaluation; three inputs for SVM-LOMETS, *i.e*. PAINT, PPA-I, and PPA-II, are components of Zhang-server, which is ranked the best among all the structure prediction servers on CASP7. Thus, the huge improvement of our method over SVM-LOMETS demonstrates that by revealing the server correlation and optimizing the gap between the true and false contacts, superior contacts can be predicted than those of other consensus methods.

### Case Study on Two CASP7 New Fold Targets

As shown in the previous section, our method significantly outperforms the other methods, especially on new fold targets. Two CASP7 new fold targets, T0319 and T0350, are investigated in this section. T0319 (PDB id 2j6a) is a zinc finger protein from the ERF1 methyltransferase complex [50] with 135 residues. T0350 (PDB id 2hc5) is protein yvyC from Bacillus subtilis [51] with 117 residues. Table 9 lists the TM-score [52], contact accuracy, and contact coverage of the best model among the five models submitted by each threading server on T0319 and T0350. All the six threading servers fail to detect correct templates. Typically, a TM-score lower than 0.17 indicates a random structure, and a TM-score higher than 0.4 indicates a meaningful structure [52]. Consequently, all the models predicted by these six servers are probably not meaningful structures.

**Table 9:** TM-score, contact accuracy, and contact coverage of the best models by the six individual servers for T0319 and T0350

		FOLDpro	mGenThreader	RAPTOR	FUGUE3	SAM-T02	SPARK3
T0319	*T Mscore*^*a*^	0.20	0.18	0.27	0.26	0.12	0.22
	*Accuracy*^*b*^	9%	9%	15%	29%	0	8%
	*Coverage*^*c*^	7%	6%	14%	13%	0	6%

T0350	*T Mscore*^*a*^	0.24	0.23	0.33	0.26	0.26	0.27
	*Accuracy*^*b*^	11%	8%	27%	9%	35%	26%
	*Coverage*^*c*^	15%	9%	29%	3%	12%	28%

^*a*^TM-score of the best model among the five submitted models for each server.

^*b*^Contact accuracy of the best model for each server. All contacts contained in this model are evaluated.

^*c*^Contact coverage of the best model for each server. All contacts contained in this model are evaluated.

**Figure 3:**
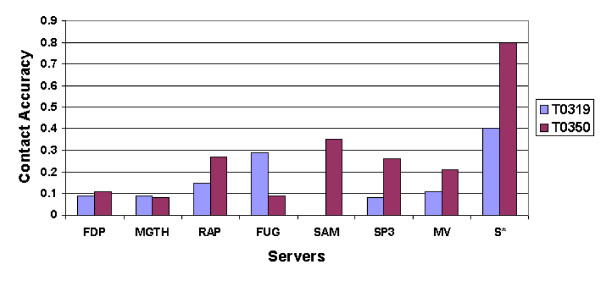
**Performance comparison on T0319 and T0350**. Accuracy of top *L*/5 contacts of the six threading servers, the majority voting method, and our method on T0319 and T0350. FDP: FOLDpro, MGTH: mGenThreader, RAP: RAPTOR, FUG: FUGUE3, SAM: SAM-T02, SP3: SPARK3, MV: majority voting, S*: our method.

The hardness of these two targets causes all the six threading servers to fail. Thus, the templates selected by the threading servers are almost random and significantly different from each other, which consequently leads to the failure of the majority voting consensus method. As shown in Figure 3, the majority voting method is even worse than some individual servers on these two targets, whereas our method performs significantly better than any individual server. In fact, the top *L*/5 accuracy of our method is 40.0% and 80.2% on these two targets, while the majority voting method achieves an accuracy of only 10.8% and 21.0%, respectively. One may argue that some of the true contacts picked up by our method are strongly supported by correlated mutation. Even when we remove correlated mutation from our method, its accuracy decreases only slightly, to 39.3% on T0319 and 79.3% on T0350. The minor difference shows that correlated mutation information is not that important for these two targets. Therefore, the case study on these two new fold targets demonstrates that by removing the server correlation and optimizing the best combination of the individual servers, it is possible to select true contacts even if the majority of the individual servers does not support them.

Although the goal of this paper is to propose a novel contact prediction method, and thus the modeling of the entire proteins is beyond the scope of this paper, it is still important to demonstrate the potential applications of the proposed method in protein structure prediction methods. One of the major bottlenecks for the state-of-the-art protein structure prediction methods is model ranking. The most widely used method for model ranking is clustering. However, although clustering based methods work well on easy and medium targets because for such targets, most of the models are high-quality ones and very similar to each other, such clustering methods usually fail on hard targets since the models usually have poor quality and are very different from each other. Thus, we test how well the contacts predicted by our method can rank the models on T0319 and T0350. We design a simple contact ranking score. Given the top *L*/5 contacts predicted by our method, for each contact, a model scores 1 if this model indeed contains this contact, and scores 0 otherwise. Table 10 shows the ranking of the best model, in terms of TM-score, of each individual server by their default model ranking method (according to the order of the models submitted to CASP7) and the ranking of the best model by our contact score. It is clear that our contact score has much better ranking of the best models for most cases. Additionally, for both T0319 and T0350, the best models generated by all these six individual servers, i.e., model 4 of RAPTOR for T0319 and model 4 of RAPTOR for T0350, are ranked first among all the models by our contact score. This demonstrates the potential applications of our contact prediction method to select better submitted models or to select good models at each iteration of the refinement process, especially for hard targets.

**Table 10:** The ranking of the best model (in terms of TM-score) for each individual server by its default ranking method and by our contact score for T0319 and T0350

		FOLDpro	mGenThreader	RAPTOR	FUGUE3	SAM-T02	SPARK3
T0319	Default ranking	4	3	4	4	4	3
	Contact ranking	2	1	1	1	3	2

T0350	Default ranking	1	3	4	4	1	4
	Contact ranking	2	1	1	1	3	1

## Discussion

The experimental results demonstrate that by accounting for the correlation among different threading programs, our consensus method can successfully identify native contacts, even when these contacts are not contained in the majority of the models. It is worth noticing that the proposed method is quite different from the more direct linear combination or non-linear combination of the original individual servers. The underlying reason is that by detecting correlation among the individual servers and removing the last latent server which corresponds to the random noise, our ILP-based optimization process is able to find an optimal solution without the bias caused by the random noise. In this paper, our method is not directly compared to other consensus contact predictors on exactly the same input servers, since servers used in other studies are not all available. Instead, six threading programs are chosen in our method. They have similar or even lower accuracy levels than those used in other consensus contact studies.

One drawback of our method is that it is a selection-only consensus method. If all the individual servers generate models with very few native contacts, our method will fail simply because there is no correct contact to choose. To avoid this drawback, a server independent feature, correlated mutation, is used to introduce some contact candidates which are not predicted by any individual server. However, the signal contained in the correlated mutation is not strong enough to find native contacts. As a result, future work will be to combine more server-independent features to introduce true contact candidates, even if all the individual servers fail to do so. Another drawback is that the small data set used in this paper makes the evaluation of the method's performance less reliable compared to more extensive tests. This is mainly due to the availability of the individual servers used in our method. We did not conduct the experiments on both CASP7 and CASP8 because some of these individual servers have been improved significantly after CASP7 and some of these servers were not available at CASP8. However, the cross-validation measurement applied in this paper can hopefully reduce the unreliability to the lowest level.

A potential application of our contact prediction method is to provide highly conserved constraints for *ab initio *folding or protein structure refinement. Recent research has shown that by incorporating contacts predicted from template-based methods or sequence-based methods, a structural model generated by comparative modeling can be refined [11,12,53,54]. However, if all the individual servers predict the structure for a target protein extremely well or very poorly, our consensus method will probably not help too much. In the former case, since almost all the contact candidates provided by these individual servers are correct ones, our method can only improve the accuracy slightly. In the latter case, since there are very few correct contact candidates for our method to choose from, the refinement process can hardly benefit from our results. However, in any other case, contacts provided by our method should help with the folding simulation. The reason is that our method can generate a small number of highly conserved contacts. Considering only a small number of contacts can reduce the conformational search space, and thus increase the speed and reduce the chance of generating wrong models. Moreover, experimental results demonstrate that our method can generate contacts with a higher accuracy than both sequence-based and template-based methods. This can reduce the risk of generating models with incorrect contacts, which can reduce the risk of selecting incorrect models from the final decoy set, and thus, greatly increase the overall *ab initio *folding accuracy.

## Conclusion

We have described a consensus contact prediction method, that is able to reduce the server correlation. Experiments on CASP7 data set show that our method significantly outperforms any previously reported study, especially on new fold targets. Therefore, the proposed method can hopefully provide a powerful tool for protein structure refinement and prediction use. The program of the proposed method is available upon request.

## Methods

Recent CASP results have indicated that correlation exists in different protein structure prediction servers, because of the common information used by the servers such as PSI-BLAST [55] sequence profile and PSIPRED-predicted secondary structure [56]. Thus, it is very likely that a true contact is supported less than some false ones due to the server correlation. Our consensus method is capable of reducing the impact caused by the server correlation. The outline of the newly developed consensus method is as follows:

• A maximum likelihood (ML) method is applied to measure the correlation coefficient between two servers.

• Principal component analysis (PCA) technique is employed to extract a few independent latent servers from a set of correlated servers.

• An integer linear programming (ILP) method is then used to assign a weight to each latent server, by maximizing the difference between the true contacts and the false ones. Also, correlated mutation is treated as a latent server which assigns a probability value to each contact candidate. This results in a consensus contact predictor that can accurately assign confidence scores to all the contact candidates.

### Notations

In this paper, a *model *refers to a protein conformation, generated by a protein structure prediction server. In contrast to human experts, a *server *refers to an automated system which predicts a set of models for a given protein (also called a *target*), whereas a *contact predictor*/*server *refers to an automated system which predicts a set of contacts. Following the contact definition used by CASPs, two residues are in contact, if the distance between their *C*_*β *_atoms (*C*_*α *_atom for Glycine), is smaller than 8Å, and they are at least six residues apart in the sequence. We call a contact *native*/*true contact*, if the two residues are indeed in contact in the native structure of the target.

The prediction accuracy is the number of correctly predicted contacts divided by the total number of contacts predicted by a predictor, while the coverage is defined as the number of correctly predicted contacts divided by the total number of native contacts. If a contact predictor is a tertiary structure prediction server, all the contacts, contained in the structural models of this server, are considered to be the contact prediction results of this server.

Let ℓ denote the number of target proteins, and *u *denote the number of input contact prediction servers. Given a target *t*_*l *_(1 ≤ *l *≤ ℓ), a server *S*_*i *_(1 ≤ *i *≤ *u*) outputs a set of models. The contacts, determined by these models, are extracted and considered as contact candidates, denoted as *C*_*i*, *l *_= {*c*_*i*, *l*, *q*_|1 ≤ *q *≤ *n*_*i*, *l*_}, where *n*_*i*, *l *_is the number of contacts, predicted by server *S*_*i *_for target *t*_*l*_. The set of all contact candidates for target *t*_*l *_is denoted as *C*_*l *_= ∪_*i*_*C*_*i*, *l*_. A consensus server aims to assign a confidence score to each candidate in *C*_*l*_.

This paper is based on the following two assumptions:

• Server *S*_*i *_generates its predictions based on a confidence measure; that is, for each contact *c *∈ *C*_*l*_, *S*_*i *_has a confidence, *s*_*i*, *c*, *l*_, on how likely it is for *c *to appear in the native structure. Since the initial confidence score is unavailable, it is approximated as follows: the number of models containing this contact divided by the total number of models generated by the server for this target.

• There are *v *implicitly independent latent servers *H*_*j *_(1 ≤ *j *≤ *v*) dominating the explicit servers *S*_*i*_. Given a target *t*_*l*_, *H*_*j *_assigns a value *h*_*j*, *c*, *l *_(*c *∈ *C*_*l*_) as the confidence score on how likely *c *is a native contact.

Identifying independent latent servers is essential to reduce the negative effects of the server correlation and to reduce the dimensionality of the search space, as the number of latent servers is expected to be smaller than the number of original servers. After deriving the latent servers, a new and more accurate prediction server S* can be designed, by an optimal linear combination of the latent servers, which for each target *t*_*l*_, assigns a confidence score to each contact candidate *c *∈ *C*_*l *_as follows:

(1)

where  is the weight of latent server *H*_*j *_in S*.

### Maximum Likelihood Estimation of Server Correlation

Let *O*_*i*, *j*, *l *_denote the overlap set of *C*_*i*, *l *_and *C*_*j*, *l*_; that is, *O*_*i*, *j*, *l *_= *C*_*i*, *l *_∩ *C*_*j*, *l*_, and let *o*_*i*, *j*, *l *_= |*O*_*i*, *j*, *l*_|. For a given target, let *p*_*i*, *j *_be the probability that a contact, returned by server *S*_*i*_, is the same as that returned by *S*_*j*_. Under a reasonable assumption that targets *t*_*l *_(1 ≤ *l *≤ ℓ) are mutually independent, the likelihood that server *S*_*i *_(1 ≤ *i *≤ *u*) generates contacts *c*_*i*, *l*, *q *_(1 ≤ *q *≤ *n*_*i*, *l*_) is

(2)

Therefore, the maximum likelihood estimation of *p*_*i*, *j *_can be calculated as follows:

(3)

In the rest of this paper, *P *denotes the matrix [*p*_*i*, *j*_]_*u *× *u*_.

### Uncovering Independent Latent Servers

Recall that on a target *t*_*l*_, *s*_*i*, *c*, *l *_and *h*_*j*, *c*, *l *_are the confidence scores assigned by server *S*_*i *_and *H*_*j*_, respectively. Since the latent servers are mutually independent, it is reasonable to assume that *s*_*i*, *c*, *l *_is a linear combination of *h*_*j*, *c*, *l*_(1 ≤ *j *≤ *v*) such that

(4)

where , 1 ≤ *i *≤ *u*, and , 1 ≤ *j *≤ *v*. Here, *λ*_*i*, *j *_is the weight, and a larger *λ*_*i*, *j *_implies there is a higher chance that server *S*_*i *_will adopt the contacts reported by *H*_*j*_.

From the correlation matrix of prediction servers *S*_*i*_, the factor analysis technique is employed to derive *λ*_*i*, *j *_and ; that is,  can be represented as a linear combination of  as follows:

(5)

where <*ω*_*j*,1_, *ω*_*j*,2_, ⋯, *ω*_*j*, *u*_> is an eigenvector of *P*^*T*^*P*.

### ILP Model to Optimally Combine Latent Servers

After deriving the latent servers *H*_*j*_(1 ≤ *j *≤ *v*), a new server S* can be constructed as an optimal linear combination of the latent servers. For each target *t*_*l*_, S* assigns a score to each contact candidate *c *∈ *C*_*l *_as in Eq. (1).

To determine a reasonable setting of coefficient , a training process is conducted on a data set , containing |*D*| training proteins, where *t*_*l *_is a training protein,  ⊆ *C*_*l *_denotes the set of native contacts, and  ⊆ *C*_*l *_denotes the set of false contacts. The learning process attempts to maximize the number of contacts that can be correctly identified by S*.

More specifically, for each target *t*_*l *_in the training data set, a score is assigned to each contact candidate by S*. A good contact predictor should assign native contacts with higher scores than those with false ones. The larger the gap between the scores of the native contacts and those of the false ones, the more robust this new prediction server is. In practice, a "soft margin" idea is adopted to take the outliers into account; that is, by allowing errors on some samples, we maximize the number of native contacts with a score that is higher than that of all the false ones, by at least a gap.

This optimization problem is formulated as an integer linear programming model. Let *x*_*p*, *q *_be an integer variable such that *x*_*p*, *q *_= 1 if and only if the native contact *p *is assigned a score higher than that of the false contact *q *by at least *ε *by the new server; *x*_*p*, *q *_= 0, otherwise. Here, *ε *is a parameter used as the lower bound of the gap between the score of a native contact and that of the false ones. Similarly, *y*_*p*, *l *_= 1 if and only if the native contact *p *has a score higher than that of all the false contacts in ; *y*_*p*, *l *_= 0, otherwise. The goal is to maximize the number of native contacts that have higher score than that of all the false contacts.

Consequently, the consensus contact prediction problem is formulated by the following ILP model:

(6)

(7)

(8)

(9)

(10)

Constraint (7) forces *x*_*p*, *q *_to be 0 if the gap between the scores, assigned to the native contact *p *and the false contact *q *, is smaller than *ε*. If a native contact *p *has a score not higher than all the false contacts, constraint (8) forces *y*_*p*, *l *_to be 0. Thus, there is no contribution to the objective function. Constraint (9) normalizes the weights, and constraint (10) restricts *x*_*p*, *q *_and *y*_*p*, *l *_to be either 0 or 1. The objective function is the number of native contacts that have higher scores than all the false contacts.

### New Prediction Server

After the independent latent servers are derived and the optimal weights are trained, a new contact predictor is formed. Given a query target *t*_*l*_, each server *S*_*i *_produces a set of contact candidates, *C*_*i*, *l*_. The set of all the candidates is denoted as *C*_*l *_= ∪_*i *_*C*_*i*, *l*_. For each contact candidate *c *∈ *C*_*l*_, the confidence assigned by the latent server *H*_*j *_is calculated by Eq. (5). Then, the new consensus server S* assigns a confidence score to contact candidate *c *according to Eq. (1). S* assigns a confidence score to each contact candidate, and picks up the top scored ones as the final predictions.

## Authors' contributions

XG and DB carried out the implementation and computational analysis. All authors participated in designing the study and preparing the manuscript. All authors read and approved the final manuscript.
